# Accelerating discovery and rational engineering of antibody modalities using Cryo- EM

**DOI:** 10.1063/4.0001139

**Published:** 2025-10-27

**Authors:** Tilak Gupta, Surajit Banerjee

**Affiliations:** Thermo Fisher Scientific, Portland, Oregon, USA

## Abstract

As the need for novel therapeutic modalities is growing, researchers require innovative strategies and transformative technologies to overcome the limitations of traditional antibody discovery methodologies. Cryo-electron microscopy (Cryo-EM) has been successfully established as a powerful technique to accelerate antibody drug discovery processes. Despite the recent adoption of cryo-EM in drug discovery efforts, multiple cryo-EM supported therapies have already entered the clinics. The broad applicability of single particle imaging underpins the rapidly increasing implementation of cryo-EM in antibody drug discovery pipelines including monoclonal antibodies, design of bi- and multi-specific formats, and design of CAR binders for CAR-T cell therapy. Here, we will provide specific and diversified applications of cryo-EM that will contribute to reshape the future of antibody research. We will share how cryo-EM based epitope mapping provide accurate information at the single amino acids. We will explain ways one can leverage cryo-EM structures to design of next-generation antibody therapeutics. We will discuss our view on how cryo- EM can provide experimental complementation to Artificial Intelligence/Machine Learning (AI/ML) tools. Finally, we will touch base to cryo-EM Polyclonal Epitope Mapping (Cryo-EMPEM), a workflow that allowed to structurally characterize immunogenicity of antibody therapeutics and vaccines.

